# Stability and Reliability of Repeated Plasma Pregnenolone Levels After Oral Pregnenolone Dosing in Individuals with Cocaine Use Disorder: Pilot Findings

**DOI:** 10.3390/life14111483

**Published:** 2024-11-14

**Authors:** Huaze Gao, Zachary Magin, Nia Fogelman, Rajita Sinha, Gustavo A. Angarita, Verica Milivojevic

**Affiliations:** 1 The Yale Stress Center, Department of Psychiatry, Yale University School of Medicine, New Haven, CT 06519, USA; huaze.gao@yale.edu (H.G.); zachary.magin@uconn.edu (Z.M.); nia.fogelman@yale.edu (N.F.); rajita.sinha@yale.edu (R.S.); 2 Clinical Neuroscience Research Unit, Connecticut Mental Health Center, Yale University School of Medicine, New Haven, CT 06519, USA; gustavo.angarita@yale.edu

**Keywords:** pregnenolone, stability, reliability, cocaine use disorder

## Abstract

Substance use disorders (SUDs), including cocaine use disorder (CUD), have significant negative health risks and impose a substantial social burden, yet effective treatments are limited. Pregnenolone, a neuroactive steroid precursor, has been shown to reduce alcohol craving and normalize stress biology in individuals with CUD, but its clinical utility has been questioned due to limited data on bioavailability and the stability of blood levels in humans. Thus, this pilot study aimed to determine whether twice-daily oral pregnenolone (PREG) at 300 mg/day and 500 mg/day versus placebo in week two of PREG administration led to stable increased plasma pregnenolone levels in individuals with CUD. Seven treatment-seeking individuals with CUD, enrolled in an eight-week double-blind clinical trial, were randomized to receive placebo (*n* = 2) or pregnenolone at 300 mg/day (*n* = 3) or 500 mg/day (*n* = 2). For the first two weeks of the eight-week trial, participants were admitted to an inpatient Clinical Neuroscience Research Unit for repeated serial sampling of plasma pregnenolone concentrations over a 32.5 h period in week two of their inpatient stay while taking their assigned study drug under observation. Pregnenolone levels showed a significant main effect of the medication group (*p* = 0.039), with sustained higher levels in the 300 mg (*p* = 0.018) and 500 mg (*p* = 0.035) groups compared to placebo, and no significant difference between the two pregnenolone dosing groups. Moreover, correlation analyses showed that after observed study medication dosing on repeated sampling day 1, levels of pregnenolone were highly associated across time, with strong, positive correlations between time of dosing and 2 h (r = 0.80, *p* = 0.031), 4 h (r = 0.80, *p* = 0.031), 6 h (r = 0.86, *p* = 0.013), and 8 h post-dosing (r = 0.97, *p* < 0.001). These findings from this pilot study suggest that chronic twice-daily/“bis in die” (b.i.d.) oral administration of pregnenolone at both 300 mg/day and 500 mg/day achieved stable and reliable elevated plasma pregnenolone levels over 32.5 h in individuals with CUD, thereby supporting the good bioavailability of pregnenolone in these samples. These data indicate that twice-daily chronic dosing may overcome any potential concerns of poor bioavailability and rapid metabolism of pregnenolone in humans, and support further clinical investigations into pregnenolone’s role in the treatment of cocaine use disorders.

## 1. Introduction

Substance use disorders (SUDs), including cocaine use disorder (CUD), are major global health concerns contributing to a wide range of medical, psychological, and social problems. In the United States, SUDs affect an estimated 48.5 million people aged 12 or older, including 1.3 million people with CUD [1]. CUD leads to complex comorbidities, such as heart attacks and strokes [2,3], and imposes significant societal costs, including increased crime rates, loss of productivity, and contributions to the illicit drug market [4,5]. Despite the profound impact of these disorders, effective treatments are currently very limited. In the US, SUD treatment engagement is very limited and there are no FDA-approved medications available for CUD, highlighting a critical need for effective novel pharmacotherapies.

Neuroactive steroids have emerged as promising potential targets in the treatments of substance use disorders due to their ability to modulate several neurotransmitter systems involved in addiction. Specifically, they modulate the hypothalamic–pituitary–adrenal axis, enhancing GABAergic neurotransmission to restore homeostasis after stress, which may prevent excessive alcohol consumption [6,7]. Pregnenolone, the precursor of many other neuroactive steroids, has attracted significant attention. Animal studies have shown that pregnenolone and its GABAergic metabolites, such as allopregnanolone, can restore normal physiological stress responses, reduce anxiety, and modulate alcohol consumption behavior [8,9,10,11]. Furthermore, recent clinical studies have extended these findings to humans with promising results. Findings from our laboratory have shown that chronic pregnenolone treatment in separate samples of individuals with alcohol use disorder (AUD) and CUD reduces cravings for alcohol and cocaine and normalizes physiological responses during experimental stress and drug cue-related provocations [12,13]. These findings suggest the potential of pregnenolone to treat SUD and improve clinical outcomes, particularly through its ability to reduce craving, a key predictor of substance use and relapse [14].

Despite this growing evidence supporting the potential of pregnenolone, some concerns about its clinical utility have been raised due to possible poor bioavailability, rapid metabolism, and instability in the body, which could limit its practical effectiveness [15]. For example, some rodent research assessing blood and brain levels after acute single intravenous and intra-nasal administration suggested rapid degradation and poor bioavailability in the brain and blood regardless of the route of administration, with steroid dehydroxylases identified as one potential reason [16]. In addition, even though early human studies showed robust increases in serum pregnenolone levels after acute doses of pregnenolone and pregnenolone sulfate [17] and significant cortico-limbic striatal activation after acute 400 mg of pregnenolone vs. placebo [18], these studies offer limited information on chronic dosing and the specific doses required for stable therapeutic levels. Thus, the concerns regarding potential rapid metabolic conversion have not been addressed, thereby posing challenges for treatment adherence and sustained treatment effects, both of which are crucial for clinical success.

To address this research gap, it is essential to determine the stability of pregnenolone levels with varying repeated doses in clinical populations. Thus, in the current pilot study, we aimed to determine whether twice-daily oral self-administration of pregnenolone at total daily doses of 300 mg or 500 mg results in stable elevated pregnenolone blood levels in individuals with CUD. Repeated serial assessments of pregnenolone levels were conducted over a 32 h period in a controlled, observable inpatient setting while patients were in the first two weeks of an eight-week treatment regimen. By monitoring plasma pregnenolone concentrations over a period exceeding 24 h, we sought to address concerns about its short half-life and metabolic stability. Our findings may inform dosing strategies and support further clinical investigations into pregnenolone’s role in treating SUDs.

## 2. Materials and Methods

### 2.1. Participants

**Figure 1:**
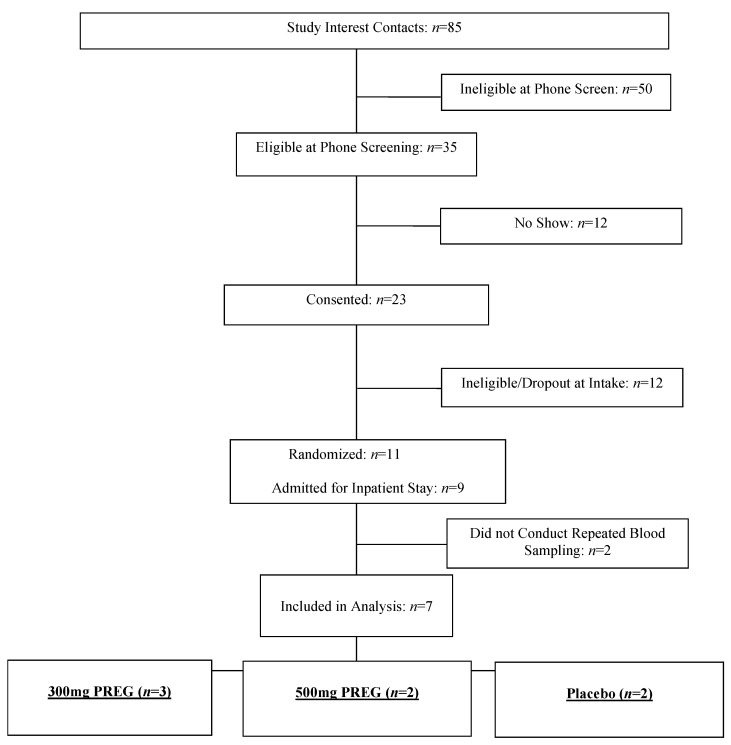
CONSORT flow diagram.

Seven treatment-seeking individuals (six males and one female) with cocaine use disorder (CUD) participated in this research, see Figure 1. Participants were recruited via local advertisements around the New Haven, CT, USA area. Current CUD was diagnosed using the Structured Clinical Interview for Diagnostic and Statistical Manual of Mental Disorders 5 (SCID-5) and confirmed with positive urine toxicology screens during the initial eligibility assessment [19]. Exclusion criteria included the following: DSM-5 substance use disorder for any psychoactive substance other than alcohol and nicotine, including opiate use disorder (such as heroin), assessed and confirmed via urine toxicology screen in addition to SCID-5; any psychotic disorder or current Axis I psychiatric symptoms requiring specific attention; and significant underlying medical conditions such as cerebral, renal, or cardiac pathology, which, in the opinion of the study physician, would preclude the patient from fully cooperating or pose potential harm during the study. Comprehensive medical assessments, including electrocardiography and laboratory tests of renal, hepatic, pancreatic, hematopoietic, and thyroid function, and a physical exam were conducted to determine eligibility. All participants provided written and verbal consent, and this study was approved by the Human Investigation Committee of the Yale University School of Medicine.

### 2.2. Study Procedures

**Figure 2:**
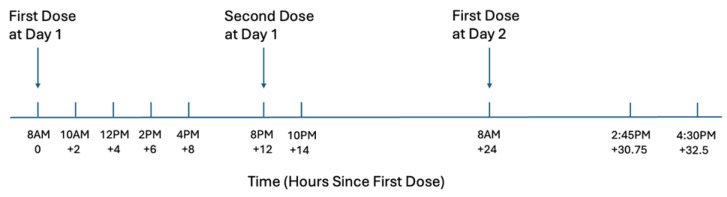
Visual timeline of study medication self-administration and corresponding serial blood sampling. In week 2 of the study medication, the first dose of the medication was administered at 8 a.m. on day 1, followed by a second dose at 8 p.m. on the same day. The first dose on day 2 was administered at 8 a.m. Blood samples were collected at various time points relative to the first dose, including immediately after (0 h), at regular intervals throughout the day (+2, +4, +6, +8, +12, and +14 h), and on the following day (+24, +30.75, and +32.5 h).

The laboratory component of this study was part of a larger eight-week clinical trial (NCT03872128). Upon determination of eligibility, participants were admitted to the Clinical Neuroscience Research Unit (CNRU) of the Connecticut Mental Health Center (CMHC) for a two-week inpatient stay in order to assess the stability of pregnenolone administration in a controlled setting. Participants were randomized to receive daily placebo (PBO) or one of two doses of pregnenolone, 300 mg/day or 500 mg/day (oral administration, in b.i.d. dosing), in a double-blind manner for a two-week inpatient phase followed by a six-week outpatient phase and continuation on the study medication for a total period of eight weeks. In the laboratory repeated serial sampling assessment, an intravenous (IV) line was placed in the participants’ non-dominant arm in week 2 of pregnenolone treatment before the 8 a.m. study medication dose. The first dose of the study medication was administered at 8 a.m., followed by a second dose at 8 p.m. on the same day. The third dose was administered at 8 a.m. on the following day. Study medication was dispensed by the research nurse on the CNRU, who also monitored the participants taking each dose. Blood collection was performed immediately after the first dose and continued up to 32.5 h post-first dose, with repeated sampling as shown in Figure 2.

### 2.3. Study Medication Dosing and Compliance/Adherence

Identical pregnenolone (150 mg and 250 mg) and placebo capsules each with 25 mg of riboflavin were prepared by the Yale University research pharmacist (Investigational Drug Services, IDS) and dispensed in 1-week bottles. We selected 300 mg/day and 500 mg/day dosing based on prior clinical trials of PREG in psychiatric patients, such as an 8-week clinical trial in patients with schizophrenia, where fixed escalating dosing from 100 mg to 500 mg of PREG [20] was well tolerated. Both participants and investigators were blinded to the treatment conditions. The Yale Stress Center Biostatistician provided medication randomization to the Yale New Haven Hospital Investigational Drug Service (YNHH-IDS) for medication formulation and dispensing while balancing for age, sex, smoking status, CUD severity, and education. During the two-week inpatient stay, pregnenolone was dispensed by the research nurse on the CNRU, who monitored the participants taking each dose. Participants self-administered the medication twice daily at 8 a.m. and 8 p.m. during the remaining six weeks of outpatient participation in the study.

### 2.4. Pregnenolone Level Measurement

Repeated blood samples were collected at multiple time points to measure pregnenolone levels. On day 1 of this serial sampling phase, blood draws were conducted at 8 a.m., 10 a.m., 12 p.m., 2 p.m., 4 p.m., 8 p.m., and 10 p.m. (0, 2, 4, 6, 8, 12, and 14 h post-first dose). On day 2, additional blood samples were collected at 8 a.m., 2:45 p.m., and 4:30 p.m. (24, 30.75, and 32.5 h post-first dose). See Figure 2 for an illustration of the blood collection timeline.

All plasma blood collection tubes were immediately placed on ice, and then centrifuged at 4 °C within 30 min of collection and immediately aliquoted into cryovials. Samples were then stored at −80 °C until processing at the Yale Center for Clinical Investigation Molecular Core Laboratories using a commercially available pregnenolone Enzyme-Linked Immunosorbent Assay (ELISA) kit (Eagle Biosciences, Inc., Nashua, NH, USA). The ELISA kit has a sensitivity of 0.05 ng/mL and a 100% specificity for pregnenolone. The coefficients of variation (CVs) of the intra-assay and inter-assay were <10.6% and <14.5%, respectively. The specificity/cross-reactivity to pregnenolone (100%) in this ELISA kit was compared to a number of other steroids, including progesterone (6%), 5α-androstanediol (4.7%), pregnenolone sulfate (0.4%), androstanedione (0.3%), DHEAS (0.2%), and several steroids for which cross-reactivity was less than 0.1% (e.g., androsterone, aldosterone, androstenedione, cholesterol, corticosterone, 5α-DHT, 17β-estradiol, and testosterone).

### 2.5. Data and Statistical Analysis

All graphing and data analyses were conducted using R v4.4.1, employing the ggplot2 v3.5.1, and dplyrv1.1.4 packages [21,22]. One-way ANOVA and chi-square tests were utilized to assess differences in demographic and cocaine use characteristics among the three participant groups. Changes in PREG concentration levels over the 24 h cycle were analyzed using linear mixed-effects models (lme4 v1.1.35.5 and emmeans v1.10.4 packages [23,24]). The models included fixed effects for the treatment groups (placebo, 300 mg PREG, and 500 mg PREG) and time points, with a random intercept for each participant. Satterthwaite’s approximation was used for the denominator degrees of freedom. The interaction term between time points and treatment was initially included but dropped due to non-significance. Pearson correlations were conducted among time points 0 to 8 h after the first dose to assess the stability of pregnenolone levels. For one subject missing the 0 h value from Day 1, the 0 h value from Day 2 was used instead, as both are 8 a.m. measurements and reflect baseline time points prior to taking the daily morning study drug. Significance for this pilot data was set at *p* < 0.05.

## 3. Results

### 3.1. Baseline Demographic and Clinical Characteristics

A total of seven participants were enrolled in this study, with five in the PREG groups (three in the 300 mg PREG group and two in the 500 mg PREG group) and two in the placebo group. The demographic and baseline characteristics of the participants are summarized in Table 1. All participants in the placebo and 500 mg PREG groups were male, while the 300 mg PREG group included two males (67%) and one female. The mean age of the participants was similar across the groups, with the placebo group averaging 50.50 years, the 300 mg PREG group averaging 52.00 years, and the 500 mg PREG group averaging 44.50 years. Other characteristics, including race, smoking status, education level, and baseline cocaine use, also showed no significant differences among the groups.

### 3.2. Plasma Pregnenolone Levels

**Figure 3:**
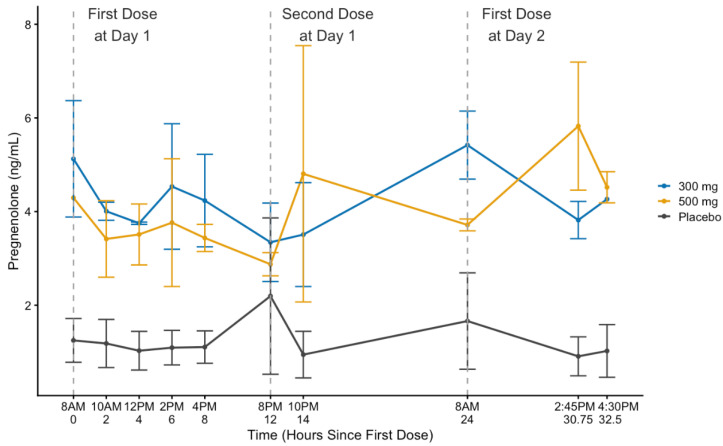
Mean plasma levels of pregnenolone, with standard error bars, for each study medication condition (pregnenolone [PREG; 300 mg, 50 mg]; placebo). Vertical dashed lines annotate timepoint immediately prior to study medication administration. Pregnenolone levels were significantly higher in both the 300 mg PREG (*p* = 0.018) and 500 mg PREG (*p* = 0.035) groups compared to the placebo, with no significant difference observed between the two pregnenolone groups (*p* = 0.686). Additionally, there was no significant main effect of time (*p* = 0.146).

There was a significant main effect of the medication group (F(2,3.924) = 8.358; *p* = 0.039) on PREG levels. Further analysis of the main effect showed that PREG levels were significantly higher in the 300 mg PREG (*p* = 0.018) and 500 mg PREG (*p* = 0.035) groups compared to PBO. No difference was observed between the two PREG groups (*p* = 0.686), suggesting that both doses achieved similar circulating levels of pregnenolone. Importantly, no significant main effect of time was observed (*p* = 0.146); see Figure 3.

### 3.3. Repeated Sampling Correlations

**Figure 4:**
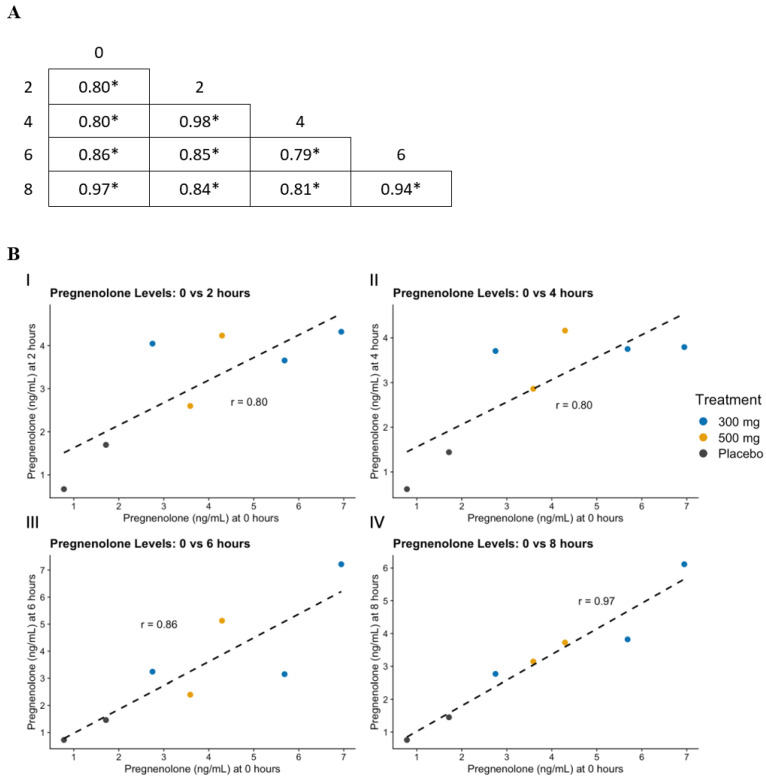
(**A**) Pearson correlation matrix of significance for levels of pregnenolone and time points. Asterisk (*) indicates positive significant correlation (*p* < 0.05). (**B**) Correlation of plasma levels of pregnenolone at time of dose one administration with pregnenolone levels at 2 h (**B**(**I**)), 4 h (**B**(**II**)), 6 h (**B**(**III**)), and 8 h (**B**(**IV**)) after pregnenolone administration.

The correlation analysis, collapsed across all three study medication conditions and conducted among repeated sampling time points 0 to 8 h following the first observed study drug administration, revealed a very high degree of association between pregnenolone levels at different time points. Specifically, strong, positive correlations were found between the pregnenolone levels at 0 and 2 h post-dosing (r = 0.80, *p* = 0.031), 0 and 4 h post-dosing (r = 0.80, *p* = 0.031), 0 and 6 h post-dosing (r = 0.86, *p* = 0.013), and 0 and 8 h post-dosing (r = 0.97, *p* < 0.001). Further details of the correlations among other time points are presented in Figure 4.

## 4. Discussion

In this study, we measured repeated pregnenolone levels during monitored, double-blind placebo-controlled randomized chronic dosing of exogenous 300 mg and 500 mg of pregnenolone and placebo administered in a b.i.d dosing schedule in individuals with CUD over a 32.5 h period. Pregnenolone, 300 mg/day or 500 mg/day, or placebo was self-administered under observation in an inpatient research unit during the second week of an eight-week clinical trial. Our findings suggest that twice-daily oral administration of pregnenolone at both the 300 mg and 500 mg doses successfully achieved elevated and stable plasma levels over an extended period, with no significant fluctuations over time, addressing some previously raised concerns about its rapid metabolism and instability.

This is the first study to assess repeated plasma pregnenolone levels after chronic and multiple doses of pregnenolone administration in humans in a randomized, double-blind design to determine its stability and reliability in support of its potential clinical utility. Previous pharmacokinetic and pharmacodynamic assessments of pregnanolone, a downstream metabolite of pregnenolone, following IV administration in eight healthy volunteers showed a terminal half-life ranging between 72 and 212 min [25]. While we were not able to assess the half-life of pregnenolone in the current study, the current findings show that the levels of pregnenolone remain elevated and stable between the 12 h dosing regimens. The absence of a significant main effect of repeated time points further supports this, suggesting no significant fluctuations in pregnenolone levels over the 32.5 h repeated sampling period in the context of b.i.d. doses. Additionally, our findings showed that pregnenolone levels were very highly correlated, and repeated 2 h sampling between dose one and dose two showed a strong relationship between pregnenolone levels across those time points, demonstrating a high level of reliability, thereby suggesting good bioavailability.

Instead of measuring the increase in pregnenolone levels after an acute dose, this study examines its stability during an ongoing chronic dosing regimen. While previous work has shown that levels of pregnenolone increase significantly in response to an acute high dose of pregnenolone [18], the time course of this increase was not fully understood. Furthermore, previous studies that examined chronic pregnenolone treatment in individuals with schizophrenia and bipolar disorder found that treatment with pregnenolone for 8 to 12 weeks significantly increased serum levels of pregnenolone [20,26,27]. However, these studies assessed pregnenolone levels once during chronic treatment compared to baseline pre-treatment levels, and our findings of repeated assessment of pregnenolone levels during a chronic treatment regimen further add to the literature by showing the stability and reliability of pregnenolone elevation with repeated chronic dosing.

The ability to maintain stable and elevated circulating levels of pregnenolone holds significant clinical implications. For individuals with CUD, this stability may result in more consistent therapeutic effects, such as reduced craving and improved physiological responses, as observed in our previous studies [12,13]. By mitigating concerns related to rapid metabolism, our study further confirms pregnenolone’s potential as a promising candidate for treating substance use disorders at the specific doses and dosing regimens tested. Additionally, the lack of FDA-approved medications for CUD underscores the necessity of exploring pregnenolone as a novel pharmacotherapy, providing a new avenue for substance use disorder treatment.

Despite the promising findings, several limitations warrant consideration. First, as this was a pilot study with only seven participants, including only one female, the small sample may limit both the statistical power to detect subtle differences between dosing groups and the assessment of potential sex-specific effects. Additionally, plasma pregnenolone concentrations were measured during the second week of the eight-week pregnenolone treatment and after participants had already been taking pregnenolone for a week rather than after the first-ever dose of pregnenolone, providing a snapshot of plasma levels rather than a comprehensive view of pregnenolone’s pharmacokinetic profile, acutely or with chronic treatment over the entire eight-week treatment period. Consequently, we lack data on other pharmacokinetic parameters, such as absorption rate and half-life. Another potential limitation is that the inpatient stay itself could have affected the observed pregnenolone levels; however, any acute effects of this should have been alleviated by conducting the repeated assessment in week two of the inpatient stay and after the participants had adjusted and stabilized. Further, any effects of the inpatient unit would be similar in the placebo and pregnenolone dose groups and would therefore be non-specific and equivalent across groups. Future studies should involve larger, more diverse populations and extend the duration of monitoring to fully elucidate pregnenolone’s pharmacokinetics.

## 5. Conclusions

Despite these limitations, our study provides preliminary evidence that twice-daily oral administration of pregnenolone achieves stable and elevated plasma levels in individuals with CUD, supporting its potential bioavailability and warranting further study of its therapeutic effects.

## Figures and Tables

**Table 1:** Demographic characteristics of the CUD sample (N = 7).

	Placebo	300 mg PREG	500 mg PREG
	(*n* = 2)	(*n* = 3)	(*n* = 2)
Male	2 [100%]	2 [67%]	2 [100%]
Age	50.50 ± 2.12	52.00 ± 4.58	44.50 ± 4.95
Race			
African American	2 [100%]	3 [100%]	1 [50%]
White	0 [0%]	0 [0%]	1 [50%]
Regular Nicotine Smoker	2 [100%]	2 [67%]	2 [100%]
Alcohol Use Disorder (AUD)	1 [50%]	2 [67%]	1 [50%]
AUD Severity			
Mild	1 [100%]	1 [33%]	1 [50%]
Moderate	0 [0%]	1 [33%]	0 [0%]
Education	10.00 ± 1.41	13.00 ± 1.73	14.00 ± 2.83
Cocaine Use Disorder (CUD) Severity			
Mild	0 [0%]	1 [33%]	0 [0%]
Moderate	1 [50%]	0 [0%]	1 [50%]
Severe	1 [50%]	2 [67%]	1 [50%]
Baseline Week Cocaine Grams	0.14 ± 0.04	0.22 ± 0.20	0.53 ± 0.66
Baseline Week Percent of Cocaine Use Days	35.71 ± 10.10	45.71 ± 32.95	71.43 ± 40.41

Note: Standard deviations are presented following ±, and percentages are indicated in parentheses. All variables: *p* > 0.05.

## Data Availability

The original contributions presented in the study are included in the article, further inquiries can be directed to the corresponding author.
